# Lexical alignment persists across a 12 h interval but is unaffected by sleep versus wake

**DOI:** 10.1038/s41598-025-33541-2

**Published:** 2026-01-22

**Authors:** Yicheng Qiu, Lewis V. Ball, M. Gareth Gaskell, Heather J. Ferguson

**Affiliations:** 1 School of Psychology, University of Kent, Canterbury, UK; 2 School of Psychology, University of York, York, UK

**Keywords:** Sleep consolidation, Lexical alignment, Social interaction, Familiarity, Human behaviour, Psychology

## Abstract

Sleep may play a key role in consolidating social interactions by transforming brief communicative experiences into stable social memories. In this paper, we used the lexical alignment task to investigate whether sleep enhances the consolidation of shared linguistic representations between partners. Lexical alignment, where participants converge their word choices with those produced by a partner, served as a marker of successful communication and shared understanding. Eighty-two participants completed a picture-matching and picture-naming task before and after 12 h of sleep or wake. Results showed the lexical alignment effect persisted 12 h after the initial picture-matching interaction. However, this effect was not influenced by whether participants slept or remained awake during the retention interval. These findings suggest that while participants encoded enduring representations of partner-specific lexical information during interaction, sleep-based consolidation did not confer an additional benefit. One possibility is that a higher level of social relevance is needed for these memories to be susceptible to sleep consolidation. Alternatively, limited hippocampal involvement during encoding may have reduced the extent to which information was reactivated during sleep. Future research should employ richer and more interactive tasks to clarify how sleep supports the consolidation of social experiences and relationship perceptions.

## Introduction

Sleep plays a critical role in supporting a wide range of cognitive functions^1,2^, including those that underpin success in our social relationships and interactions^3^. Successful social interactions often depend on our ability to remember specific details from past encounters—what was said, how someone behaved and how we felt^4,5^. It has been proposed that sleep may play a key role in preserving social episodic memories, for example details of a conversation, particularly when they carry emotional or personal significance^6–8^. Over time, sleep may transform detailed social memories into more abstract representations, or “gist” memories, which capture enduring traits, relationships or intentions of others^9,10^. In this paper, we investigate whether sleep can enhance the encoding and consolidation of social information by examining how a period of sleep *versus* wakefulness influences subsequent interactions between previously unacquainted partners.

A key mechanism through which sleep influences cognitive functioning is episodic memory—a form of memory that allows individuals to recall specific events, enriched with contextual details about what happened, where and when. Episodic memory relies on a network involving the hippocampus and pre-frontal cortex and is particularly susceptible to interference from new information^11^. Without active rehearsal or elaboration, episodic details often fade over time. However, these memories can be stabilised into longer-term memory stored during sleep, a process known as sleep-related memory consolidation. During slow-wave sleep, episodic memories are replayed in the hippocampus and gradually integrated into cortical networks through the refinement of synaptic connections^12–14^. While the role of sleep in consolidating episodic memory is well established in cognitive domains such as learning and language^15–18^, its potential to support the consolidation of social memories and experiences remains less well understood. This gap is important because episodic memory is an essential element of social cognition^7,9,10^.

In this paper, we test the idea that sleep-dependent memory consolidation could influence how we behave in future interactions with the same individual, contributing to the development of social understanding and relationship formation. We focus on a concrete behavioural outcome that reflects the integration of memory and social cognition: alignment in language use. According to Pickering and Garrod’s^19^ interactive alignment model, successful conversation involves the dynamic coordination of meaning between speaker and listener, as they converge on shared representations and mutual knowledge^20,21^. This coordination is made possible by the use of implicit social memory—an evolving representation of what is jointly known—which guides language choices and interpretation^22^. Shared knowledge shapes behaviour in subsequent interactions, as can be seen, for example, in partner-specific conceptual pacts^23–25^. Alignment manifests at multiple levels, from shared lexical and syntactic choices^26,27^ to the mirroring of gestures and gaze^28^.

Recent research suggests that people form episodic contextual representations of words in sentences, and that these memories influence later lexical processing^18,29,30^. However, it is unclear whether alignment might be supported by similar episodic memories of past language use. It is possible that episodic memories of language used in conversation could affect later lexical choices, and may be subject to the same sleep-dependent consolidation processes that have been observed in other memory domains^17,18,29,30^. Thus, encoding and retrieving shared language experiences could depend on hippocampal memory systems^31,32^, offering a promising context in which to examine how sleep impacts the consolidation of socially-relevant episodic memories.

To date, alignment has been widely studied in dyads of unfamiliar participants, and relatively little work has explored how the social relationship between interlocutors affects alignment. One exception is Riordan et al.^33^, who found that friends show greater alignment than strangers across multiple dimensions, including semantic and affective content. This suggests that closer social bonds enhance conversational coordination, potentially due to richer shared knowledge. Other work has shown that feelings of social exclusion increase syntactic alignment, indicating that alignment can be modulated by social context^34^. Together, these findings underscore the role of social variables in shaping alignment processes.

The current study examines whether sleep can enhance the consolidation of shared social experiences between previously unacquainted conversational partners. We test whether a period of overnight sleep, compared to wakefulness, influences alignment in a subsequent conversation and is associated with changes in perceived social connection. In doing so, we aim to bridge literatures on sleep, episodic memory, language processing and social interaction—highlighting sleep as a potential mechanism for strengthening the foundations of social communication.

## The current study

We chose the lexical alignment task^27^ to assess sleep-dependent consolidation of social episodic memory because it is a well-established measure of how interlocutors adapt to each other’s linguistic choices during communication^23,25^. In this task, participants complete a picture-matching and picture-naming task with a partner (in fact, partner responses are computer-generated). On picture-matching trials, the participant sees an object name (which they believe was typed in by their partner) and must choose the matching image from a pair. On picture-naming trials, the participant must label one of two images so that their partner can select the correct match. Critically, experimental trials include images that can be labelled with either a favoured or a disfavoured name (e.g. *basket* and *hamper*, respectively), allowing us to measure how often participants adopt their partner’s lexical choices (i.e. align to use the same label on picture-naming trials as their partner did on a previous picture-matching trial of the same image). Branigan et al.^27^ found that participants reliably repeated their partner’s lexical choice, including disfavoured names, indicating a robust tendency toward alignment. Importantly, this alignment reflects encoding of the partner’s lexical choices in memory, which can be considered a form of social-episodic memory. According to Gaskell’s^35^ dual-pathway model of lexical processing, recently experienced linguistic items are initially supported by flexible hippocampal pathways that can be reactivated during sleep. Although this model does not explicitly consider partner-specific memory effects, any component of a linguistic episode, including partners in discourse, could conceivably be represented in the hippocampal pathway, such that the memory of these partner-specific associations is strengthened during sleep. In the present study, we therefore tested whether this tendency to align, especially on disfavoured terms, was stronger after a period of sleep compared to wakefulness, and whether any changes were associated with enhanced perceptions of social connection (i.e. familiarity, common ground and likeability).

Participants completed the lexical alignment task across two lab sessions, separated by 12 h, either including a night of sleep or a day of wakefulness. In the sleep condition, participants completed Session 1 at 8:00 PM and returned after a night of sleep for Session 2 at 8:00 AM. In the wake condition, participants completed Session 1 at 8:00 AM and returned that evening for Session 2 at 8:00 PM, after remaining awake. This design allowed us to test whether alignment effects and social relationship measures differed following a period of sleep *versus* wake.

To promote initial social engagement between participants, each pair completed a 5 min face-to-face problem-solving task^36^ prior to the alignment task in Session 1. The alignment task was then completed in separate, adjacent cubicles to standardise the interaction setting. In both sessions, participants also completed self-report measures of their perceived relationship with their partner. These included ratings of familiarity^37^, perceived common ground^38^ and likeability (Reysen Likeability Scale^39^, enabling us to examine whether social closeness changed across sessions and whether this change differed by sleep condition.

To rule out pre-existing differences in sleep patterns or sleepiness between groups, we also collected baseline sleep measures during Session 1. These included the Pittsburgh Sleep Quality Index (PSQI^40^, the Epworth Sleepiness Scale (ESS^41^, the reduced Morningness and Eveningness Questionnaire (rMEQ^42,43^, and the Stanford Sleepiness Scale (SSS^44^.

### Hypotheses

1. Given the random assignment of participants to Sleep and Wake groups, we predicted no significant difference between groups in longer-term sleep quality or circadian preference measures, as measured by the PSQI, ESS and rMEQ.

2. We predicted that all participants would report improved social relationship quality in Session 2 compared to Session 1, due to the mere passage of time and implicit memories of the interaction experience. Critically, we expected that participants in the Sleep group would show a greater increase in perceived familiarity, common ground and likeability than those in the Wake group. This prediction is based on studies showing that sleep-dependent consolidation (i.e. the reactivation and consolidation of social experiences during sleep) strengthens both the informational and affective representations of prior social interactions^8,45,46^, which may lead individuals to experience greater familiarity and rapport with a partner upon re-engagement.

3. We predicted that lexical alignment—measured as the tendency to repeat a partner’s lexical choices—would decline from Session 1 to Session 2 due to the passage of time since the lexical prime. However, we expected this decline to be smaller in the Sleep group than in the Wake group, consistent with sleep-dependent consolidation of conversational memory. If a sleep effect on alignment was observed, we also planned to explore whether post-sleep increases in perceived social closeness (i.e. familiarity, likeability and common ground) mediate the effect.

4. Based on prior evidence linking sleep quality to social outcomes^47^, we predicted that individual differences in baseline sleep quality would correlate with social relationship ratings and alignment in Session 1. Specifically, participants reporting higher sleep quality (i.e. lower PSQI, ESS and SSS scores) were expected to show stronger perceived connection with their partner and greater lexical alignment.

## Results

All datasets and analysis scripts are available in the Open Science Framework repository, https://osf.io/s3cpz. We note some minor deviations from the pre-registered analyses: t-tests were replaced with linear mixed effects models to analyse sleep quality data; item was included as a random effect in generalised linear mixed effects models of lexical alignment. A summary of descriptive statistics is presented in Table 1.

**Table 1:** Summary of sleep quality measures, social outcome measures, and the proportion of disfavoured target responses in the lexical alignment task.

		Wake		Sleep	
		Session 1	Session 2	Session 1	Session 2
Sleep measures	PSQI (max. 21)	7.95 (3.31)	–	8.43 (3.15)	–
	ESS (max. 24)	7.33 (3.67)	–	7.85 (3.70)	–
	rMEQ (max. 25)	11.67 (3.59)	–	12.03 (3.71)	–
	SSS (max. 7)	3.07 (1.26)	3.10 (1.59)	2.68 (0.80)	2.98 (1.37)
Social outcome measures	Familiarity (max. 5)	2.10 (0.98)	2.57 (0.97)	1.88 (0.85)	2.43 (0.84)
	Common Ground (max. 40)	24.33 (4.88)	24.71 (4.70)	23.33 (4.90)	23.08 (4.11)
	Likeability (max. 77)	57.52 (7.35)	55.71 (7.61)	55.60 (7.27)	54.13 (7.69)
Lexical alignment task (proportion of disfavoured name responses)	Favoured name prime	0.01 (0.03)	0.01 (0.04)	0.01 (0.02)	0.03 (0.05)
	Disfavoured name prime	0.35 (0.26)	0.2 (0.19)	0.33 (0.25)	0.19 (0.17)
	Alignment effect	0.34 (0.27)	0.19 (0.2)	0.33 (0.25)	0.16 (0.18)

The alignment effect is the difference between the favoured and disfavoured prime conditions. Numeric values show the mean in each condition, and SDs are in brackets.

*PSQI* Pittsburgh Sleep Quality Index, *ESS* Epworth Sleepiness Scale, *rMEQ* reduced version of Morningness-Eveningness Questionnaire, *SSS* Stanford Sleepiness Scale.

### Sleep measures

A series of linear mixed effect models (using the *lme4* package in R^48^) compared sleep quality measures between Wake and Sleep groups. As expected, no significant between-group difference was found on any sleep quality measure (PSQI: β = -0.473, SE = 0.713, *t* = -0.663, *p* = .509; ESS: β = -0.517, SE = 0.813, *t* = -0.635, *p* = .527; rMEQ: β = -0.358, SE = 0.806, *t* = -0.445, *p* = .658). SSS did not differ between Sessions 1 and 2 (β = 0.162, SE = 0.196, *t* = 0.827, *p* = .411), and the effect of Group was not significant in either Session (Session 1: β = -0.396, SE = 0.234, *t* = 1.696, *p* = .094; SSS in Session 2: β = 0.120, SE = 0.328, *t* = 0.366, *p* = .715).

### Social outcome measures

To test whether sleep enhances the quality of social relationships between strangers, three generalised linear mixed effect models were run on each of the social outcome measures (familiarity, perceived common ground and likeability). Group (Wake vs. Sleep) and Session (1 vs. 2) were included as fixed factors using sum coding (− 0.5 vs. 0.5), alongside a random intercept for participants. The models used a BOBYQA optimizer with a set maximum of 100,000 iterations to increase chances of convergence.

Familiarity increased significantly from Session 1 to Session 2 (β = 0.558, SE = 0.110, *t* = 5.083, *p* < .001), but did not differ between groups (β = 0.097, SE = 0.196, *t* = 0.492, *p* = .623) or interact with Group (β = − 0.414, SE = 0.220, *t* = − 1.883, *p* = .060). Exploratory post-hoc analyses of the marginal interaction suggested that the increase in familiarity from Session 1 to 2 was larger in the Sleep group (β = − 0.765, SE = 0.157, *z* = − 4.863, *p* < .001) than the Wake group (β = − 0.351, SE = 0.153, *z* = − 2.289, *p* = .022). Perceived common ground was not affected by Session, Group or their interaction (all βs < 1.378, *p*s > 0.292). Likeability decreased significantly from Session 1 to Session 2 (β = − 1.742, SE = 0.304, *t* = − 5.740, *p* < .001), but did not differ between Groups (β = 1.738, SE = 2.223, *t* = 0.782, *p* = .434), nor did the Session effect interact with Group (β = − 0.313, SE = 0.607, *t* = − 0.516, *p* = .606).

### Lexical alignment task

Following Branigan et al.^27^, the dependent variable was a binary value that coded whether or not participants aligned with the name used by their “partner” on a preceding matching trial of that same image (i.e. used a disfavoured word following a disfavoured matching trial or a favoured word following a favoured matching trial; coded as 1 or 0, respectively).

First, we used a Wilcoxon signed-rank test to test whether the alignment effect was significantly larger than zero (i.e. no alignment) in each session. This confirmed that the lexical alignment effect was significantly above zero at both Session 1 (V = 2693, *p* < .001) and Session 2 (V = 1892, *p* < .001), confirming the expected tendency for participants to align their lexical choices to those produced by their partner in both sessions.

Next, to test whether sleep influences the tendency to align to the lexical choices of a conversation partner, a logit mixed-effect model was run on the words produced in the naming trials of the lexical alignment task. Group (Wake vs. Sleep) and Session (1 vs. 2) were included as fixed factors using sum coding (-0.5 vs. 0.5), alongside random effects of participants, items, lists and sub-lists. First, we constructed a logit mixed-effect model with the maximal model^49^ and used the *Buildmer* package in R (2.11^50^) to find the parsimonious random effects model. The final model only included random intercepts of participants and items, and used a BOBYQA optimizer with a set maximum of 200,000 iterations to increase chances of convergence. This analysis revealed that while the lexical alignment effect reduced from Session 1 to Session 2 (β = -0.418, SE = 0.778, *z* = -5.374, *p* < .001), it did not differ between Group (β = 0.003, SE = 0.106, *z* = 0.025, *p* = .980), nor did Session effect interact with Group (β = 0.048, SE = 0.155, *z* = 0.309, *p* = .757).

Finally, we conducted two post-hoc norming studies to verify the naming preferences for experimental images in our population. In one task, 12 new participants were shown the 18 experimental images individually and were asked to provide a name for each object by typing it in a text box (i.e. similar procedure to naming trials but with a single image stimuli and no preceding ‘prime’ name). In the second task, 12 new participants were shown the 18 experimental images individually and were asked to select which one of two names (i.e. the favoured and dispreferred names used in the current experiment) was the best fit for this object. In the open-ended naming task, participants produced the favoured name on an average of 85% of trials and the disfavoured name on 3% of trials. In the forced-choice naming task, participants selected the favoured name on an average of 85% of trials and the disfavoured name on 15% of trials. The use of a disfavoured name in our lexical alignment task was higher both in Session 1 and 2 compared to the open-ended naming task (34% vs. 20% vs. 3%, respectively). This indicates that participants did align to use the disfavoured name when their partner used this on matching trials and that this lexical alignment effect was carried over (though reduced in size) 12 h later.

### Associations between measures

**Fig. 1:**
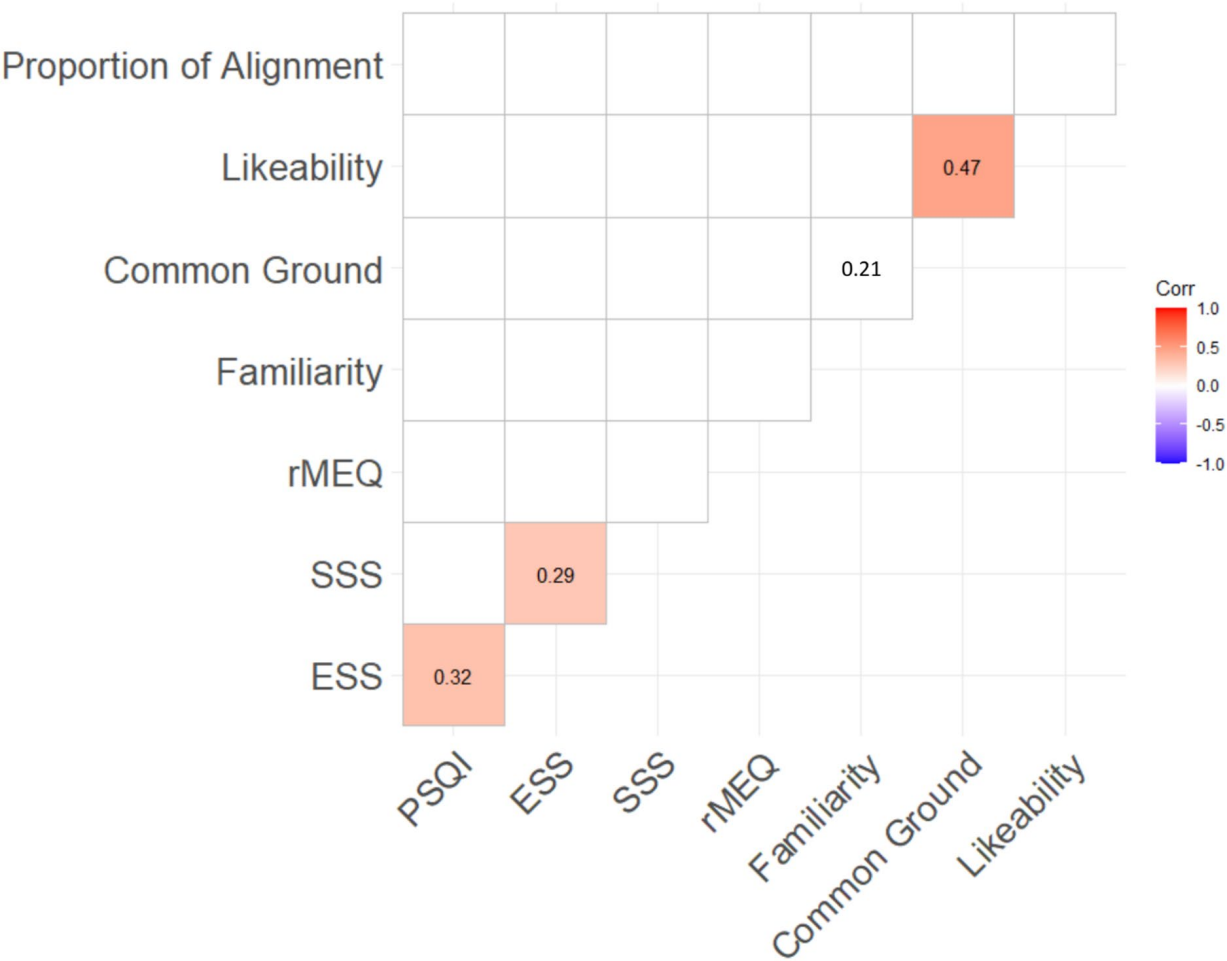
Correlation matrix between sleep quality, social outcome measures, and lexical alignment. Values show significant Spearman’s rank correlation coefficients (where *p* < .05); coloured cells show correlations that remain significant with FDR correction.

A series of non-parametric correlations (Spearman’s rank) examined associations between measures of sleep quality (PSQI, ESS, SSS, and rMEQ), social outcome (familiarity, perceived common ground, and likeability), and overall lexical alignment at Session 1. Given the number of variables included in the correlation, we applied a False Discovery Rate (FDR) correction using the Benjamini-Hochberg procedure to control for Type I error inflation^51^. This method adjusts *p*-values to account for multiple comparisons while maintaining statistical power, reducing the likelihood of false-positive findings. The resulting correlation matrix is plotted in Fig. 1.

After applying FDR correction, three correlations remained statistically significant at *p* < .05, indicating that these associations are unlikely to be false discoveries. Increased ratings of perceived common ground were associated with higher likeability ratings, and higher reported levels of daytime sleepiness (measured via ESS) were associated with both poorer sleep quality (measured via PSQI) and higher levels of current sleepiness (measured via SSS). None of the sleep quality measures correlated with social outcome measures or lexical alignment.

## Discussion

In this paper, we examined whether sleep can enhance the encoding and consolidation of social aspects of communication in a similar way to the sleep-related benefits that have been found in more cognitive domains of learning, memory and language. We used an established conversation task to measure participants’ tendency to align their lexical choices to another person and assessed the quality of the social relationship between these pairs of participants using self-report questionnaires before and after a period of sleep vs. wake. Groups were matched on their baseline levels of sleep quality and circadian rhythms (which indicated typical sleep durations^52^ in both groups) (The current study did not include any explicit measures of sleep length or quality in the intervening night between sleep group testing sessions, however participants in the sleep group were instructed to have a ‘normal’ night of sleep and participants in the wake group were instructed not to take a nap.), and current sleepiness did not differ across groups or sessions.

The primary finding is that there was no significant evidence that sleep influenced participants’ communicative behaviour in the lexical alignment task. Participants in both the sleep and wake groups spontaneously aligned to their partner’s lexical expressions, replicating the pattern reported previously^27^; they were significantly more likely to use a disfavoured object name when their partner used this name on a previous trial, in both Session 1 and 2. However, contrary to hypothesis 3, neither the size of this lexical alignment effect nor its rate of decrease from Session 1 to Session 2 differed between sleep and wake groups. Likewise, contrary to hypothesis 4, the magnitude of the lexical alignment effect in Session 1 did not correlate with any of the sleep quality measures. These findings indicate that the tendency to align lexical choices with a partner did not differ depending on whether participants stayed awake or slept after the initial encoding session. In addition, lexical alignment at Session 1 (i.e. the initial learning phase) did not differ between participants tested in the morning (AM) or evening (PM) (alignment = 0.34 vs. 0.33, respectively). This suggests that lexical encoding strength was comparable across groups, and thus any subsequent group differences (or lack thereof) cannot be attributed to baseline differences in initial lexical learning or engagement, including time-of-day effects.

The fact that alignment effects persisted in Session 2 shows that the key object names were encoded in some form of enduring memory in Session 1. However, they may not have been tagged as being communicatively relevant and this may have limited their susceptibility to sleep consolidation effects. Lexical alignment involves implicit learning of an interaction, based on memory of a single, specific event (i.e. a word prime) without a context that identifies this encoded memory as important to the individual or relevant for their future behaviour. Recent proposals that have linked sleep to social memory^7^ suggest that sleep-related memory consolidation operates by abstracting and generalising single episodic memories to new gist knowledge^10^ and prioritises information that directly supports relationships and interpersonal communication^7^. Thus, if the partner context was not sufficiently salient it would be unlikely to be prioritised for consolidation, and the detail of the social context may be abstracted out of the encoded memory.

It is possible that the procedures of our lexical alignment task were not well suited to elicit the encoding and/or consolidation of social episodic memories during sleep. The lexical alignment task provided an artificial and limited social interaction for participants, meaning that important social elements were not available or selected for encoding. Recall that in our task, interlocutors believed they were communicating with each other through separate computers by typing and receiving object names on-screen, though in reality this interaction was scripted and computer-generated. This design replicates that used in most previous studies of interactive alignment^53^ and ensures that lexical primes were properly controlled and counterbalanced, however communication did not involve the multimodal input that is typical in natural conversation (e.g. verbal, gestural, eye contact^19,28,54^. This also meant that partners could not coordinate with each other, using grounding or discussion, to mutually agree name labels for each item and this may have weakened the perception of *shared* social memories that were encoded^20,21^. Further work is needed using different tasks that provide a more interactive communication context to test the effects of sleep on social memory and communication. In addition, future work should include a control task that is known to elicit sleep consolidation effects in the cognitive domain^18,55^ to validate the sleep manipulation in the absence of effects in the social domain.

Alternatively, the lack of sleep consolidation effects may be because the lexical alignment task did not activate sufficiently high-level integration to elicit hippocampal activity during encoding, and this limited the extent to which information was reactivated during sleep. While it has been proposed that the hippocampus may be important for the flexible use and integration of language^31^, neuroimaging work has found that language alignment tasks elicit activation in classic language brain areas, including the left inferior frontal gyrus (Broca’s area), left middle temporal gyrus, and the angular gyrus^56–58^. If the hippocampus is not substantially involved in generating a contextually rich episodic memory of the object name during matching trials in the current task then what kind of memory could support the enduring alignment effect found in our study, remaining for at least 12 h? One possibility is that direct and lasting adjustments are made to the cortical networks that support object naming, meaning that there is no need for sleep to incorporate new hippocampal memories. This interpretation would be similar to the “immediate adjustment” account of word-meaning priming for lexically ambiguous words, as described by Gaskell et al.^17^. Word-meaning priming involves an exposure phase in which a lexically ambiguous word is presented in a sentence that resolves the ambiguity in favour of the less common subordinate meaning (e.g. “A pen was used by the farmer to enclose the stock before he moved them to the market.”, where *pen* is ambiguous between an animal enclosure and a writing implement). This kind of exposure led to an increased propensity to retrieve the subordinate meaning when later presented with the same word in a test phase. Importantly, word-meaning priming does appear to show sleep effects^17,30^ and so the immediate adjustment interpretation was ruled out in favour of a more episodic account.

Given the parallels between word meaning priming and the alignment paradigm studied here, it is perhaps surprising that the current study did not show significant sleep effects. One possibility is that a sentence context is needed in order to generate a contextually rich episodic memory during language exposure. If so, then the single words used here would not be sufficient. A second possibility is that the repeated nature of the alignment test in the current study (i.e. the same experimental items were tested on picture-naming trials in Session 1 and 2) may have eliminated the need for sleep in order to consolidate the priming found in the first session. Importantly, Gaskell et al.’s^17^ main results were based on a design in which items were only tested once, either soon after exposure or following a delay. They also included an analysis of items that were tested both soon after exposure and after a delay, and for these items they found equivalent priming after sleep and after wake. This was explained in terms of the potential for retrieval practice to induce a form of consolidation, meaning that sleep soon after exposure was no longer necessary to retain the priming effect^59^. A similar interpretation could be applied to the current results.

Nevertheless, our results provide the first evidence that people’s language choices continue to be influenced by lexical expressions that were used in conversation at least 12 h previously, though alignment diminishes over this period. Lexical alignment has primarily been studied as a short-term phenomenon, observed during ongoing conversations; very little is known about the duration of these effects between conversations. This finding therefore builds on research that has examined language used *within* a conversation and found that syntactic alignment effects decay rapidly as the delay between the initial message and response increases^60–62^, but can persist for much longer message-response lags (up to ten intervening filler trials) when the lexical content of the verb boosts the priming effect^63,64^ or when repeated syntactic structure primes the same verb 12 h later^65^. Since our participants were exposed to lexical stimuli in Session 1 as both a matcher and a director, it remains unclear whether the alignment effect seen in Session 2 is driven by alignment with lexical expressions used by the other person, the self or both.

Social experiences are likely encoded through both episodic and implicit forms of memory, including more implicit representations of social impressions. Our results provided limited evidence that the perceived quality of the social relationship between pairs of participants improved over time. Participants rated their partner as more familiar in Session 2 than Session 1, but perceived common ground remained stable and likeability ratings decreased across sessions. Previous research has shown that likeability ratings can be influenced by a partner’s tendency to align lexical choices with one’s own^66^. In the current study, however, stimuli were carefully randomised such that images always appeared in the matching condition first, which prevented participants from being influenced by their partner’s alignment behaviour. Crucially, none of the three social outcome measures tested here showed a sleep-dependent consolidation effect and none correlated with self-reported sleep quality. This contrasts with previous work that has linked sleep quality to longer-term social outcomes^3^, including interpersonal conflict, empathy, satisfaction in social relationships, and emotional processing^67–72^. In fact, this relationship is bidirectional: negative social experiences can also disrupt sleep quality^47^. In our study, however, sleep quality was not associated with short-term social outcomes in interactions with a previously unfamiliar partner. It is likely that more time is needed to establish a meaningful social connection and form an episodic or implicit memory of the interaction. Only with such consolidation could sleep potentially influence affective impressions and social gist knowledge (e.g. beliefs, impressions and stereotypes) that can guide future behaviour.

In conclusion, we investigated whether sleep enhances the encoding and consolidation of social processing and did not find conclusive evidence of this relationship. Aligned lexical terms were clearly encoded in Session 1, as they persisted to influence Session 2 lexical choices. However, sleep did not influence lexical alignment behaviour in a verbal interaction or the quality of the social relationship between pairs of strangers, though these measures showed some changes over time. These findings suggest that while language alignment effects can persist for at least 12 h after encoding, sleep does not selectively consolidate either episodic retrieval of shared referential experiences or implicit social impressions of the interaction over this period relative to wake. We propose that future work adopts more interactive communication tasks that may encourage the formation of socially-relevant episodic memories, which in turn may be more susceptible to sleep-related memory consolidation.

## Methods

### Participants

Eighty-two participants were recruited from the student population at the University of Kent (70 females and 12 males; M_age_ = 19.5 years old; 42 in the wake group, and 40 in the sleep group). All were aged over 18, were native English speakers, and had normal or corrected-to-normal eyesight. All self-reported that they did not have a neurological or developmental disorder (e.g. autism), diagnosed sleep disorder, and were not currently taking any psychiatric or sleep-related medication. As recommended by Simonsohn^73^, this sample size was chosen to be 2.5 times that of Branigan et al.’s^27^ original study using this lexical decision task: Branigan et al.^27^ tested 16 participants in each treatment group, therefore, we aimed for 40 participants for each consolidation condition (Wake vs. Sleep). This sample size is also comparable to other well-powered sleep consolidation studies^17^.

Participants were tested in pairs, ensuring that pairs did not already have a social relationship prior to the experiment. Pairs of participants attended the experiment in two sessions, with a 12-hour lag between sessions to create two consolidation conditions: wake (Session 1 at 8 am and Session 2 at 8 pm, i.e. no sleep between two sessions) and sleep (Session 1 at 8 pm and Session 2 at 8 am, i.e. overnight sleep between two sessions). Participants received course credits or a cash payment for completing the experiment. All participants gave informed consent in Qualtrics. They were aware that their data would be treated confidentially and any publication resulting from this work would report only data that does not identify them. They were informed that their anonymised responses may be shared with other researchers or made available in online data repositories. The study was approved by the Ethics Committee of the School of Psychology at the University of Kent (202316996054618660) and conducted in accordance with the approved guidelines.

### Sleep measures

The quality of participants’ sleep was measured using four established self-report questionnaires. The Pittsburgh Sleep Quality Index (PSQI^40^) assessed sleep quality and disturbances over the last month using 19 self-rating questions on sleep habits. Scores were summed across items to yield a global score, ranging from 0 to 21, with higher scores indicating poorer sleep quality. The Epworth Sleepiness Scale (ESS^41^) asked participants to rate their likelihood of dozing under specific situations (e.g. when sitting and reading, when watching TV, when talking to someone, etc.). Sum scores ranged from 0 to 24, with higher scores indicating higher chances of dozing. A reduced version of the Morningness-Eveningness Questionnaire^42^, adapted from the complete version^43^, consisting of five questions, was used to assess morning/evening preference. A total score (ranging from 5 to 25) was calculated, with higher scores indicating a “morning” type, and vice versa. Since PSQI, ESS and rMEQ assess current/stable indicators of sleep, they were presented only at the beginning of Session 1. The Stanford Sleepiness Scale (SSS^44^) assessed participants’ current level of sleepiness, using a 7-point scale, where 1 is “awake” and 7 is “dream-like”. As the SSS is suitable for repeated use^74^, it was used in both Session 1 and Session 2.

### Social outcome measures

The social relationship between pairs of participants was measured in Session 1 and Session 2 using a series of rating scales. Participants rated their level of familiarity with their partner^37^ using a single question asking, “How familiar do you think you and your partner are today?” and a 5-point Likert scale (1 being “Not at all” and 5 being “Close friends”). Perceived common ground was assessed using a posterior questionnaire^38^ in which participants responded using a 10-point Likert scale to four questions on how similar or different they think they are with their partner, how much general knowledge or cultural knowledge they share, and how well they would get along with their partner if they met outside the study. Finally, likeability was assessed using the Reysen Likeability Scale^39^ in which participants rated 11 questions on the likability of their partner using a 7-point Likert scale. For all social outcome measures, a higher sum score indicated a more positive social relationship between the pairs.

### Lexical alignment task

Participants completed a lexical alignment task^27^ in both Session 1 and Session 2. This task aims to test whether participants align their lexical choices to use a word label that has been recently used by their partner, even if it is a disfavoured label for that object. Participants were told that they would complete a picture-matching and picture-naming game with a partner in a neighbouring lab cubicle, via network connected computers. Participants believed that they were interacting with the person in the next cubicle, however, all “partner” responses were scripted to simulate an interaction and were in fact computer-generated. This allowed us to properly balance the presentation of favoured and disfavoured object names for experimental trials, and replicates the approach taken in previous studies using this paradigm^27,60^. Trials alternated between selecting a picture that matched a name displayed on the screen (apparently given by their partner; referred to here as picture-matching trials), and naming pictures (referred to as picture-naming trials).

**Fig. 2:**
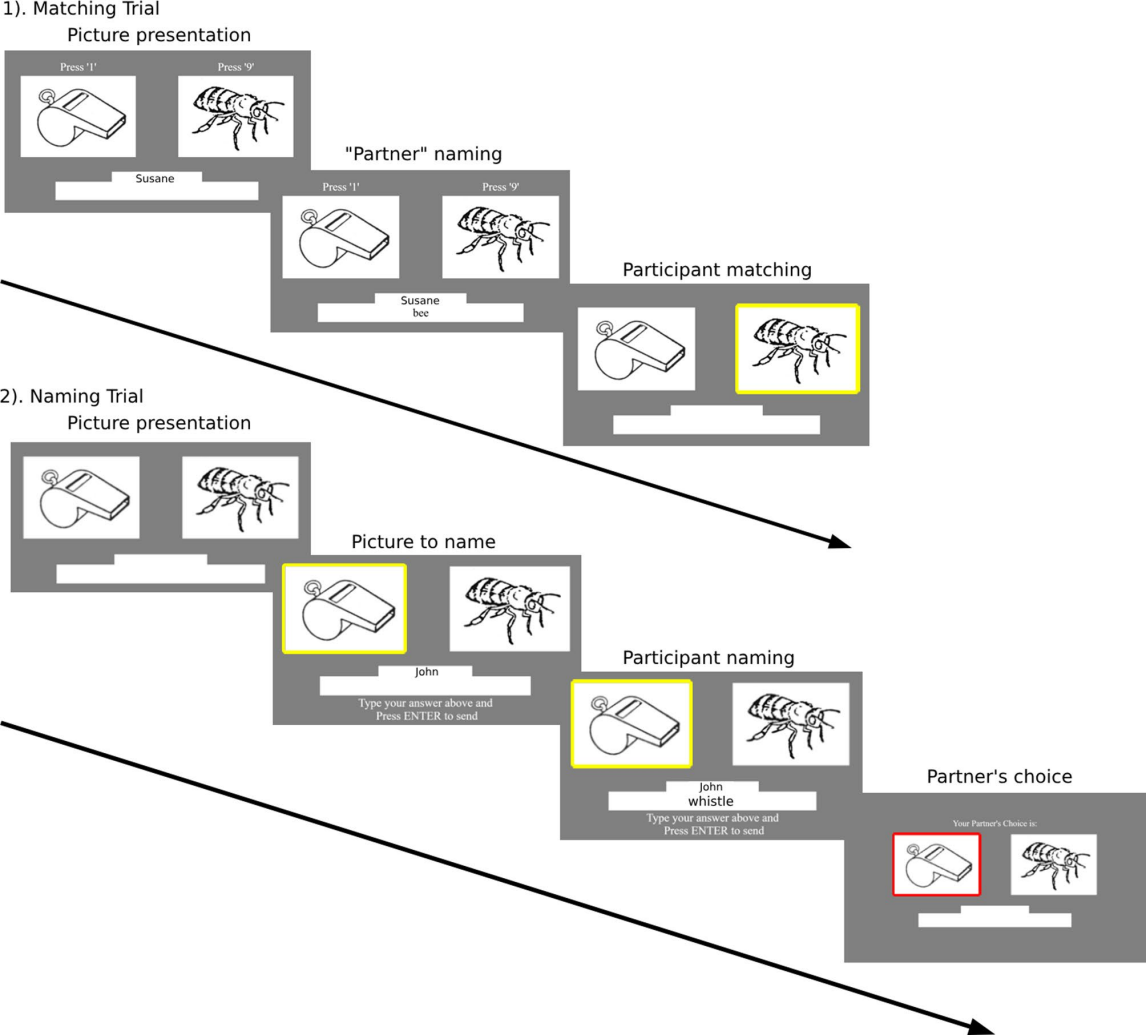
Sequence of events within a trial for picture-matching and picture-naming trials, as seen by the participant. John and Susane are used as examples of presenting real names in the experiment.

In a picture-matching trial, two images were displayed side by side with a textbox below indicating their partner’s name. After a variable delay (experimental trials for 5000 ms, filler trials for 4000 ms, 4500 ms or 5000 ms), a name for the target object ‘produced by their partner’ was shown on the matcher’s screen. Participants were instructed to select the named image using the ‘1’ and ‘9’ keys for the left or right image, respectively. In a picture-naming trial, participants were shown two images side by side. A yellow rectangle appeared around one of the images after 2000 ms. Participants were instructed to type the name of the highlighted picture into the textbox, and press ENTER. They were led to believe that this would send the response to their partner. A message, “Your Partner’s Choice is”, then appeared at the top of the screen, and a red rectangle was displayed after 1500 ms or 2000 ms to indicate their partner’s choice of image based on their typed answer. Figure 2 illustrates the sequence of events, as seen by the participant, within a picture-matching and picture-naming trial.

**Fig. 3:**
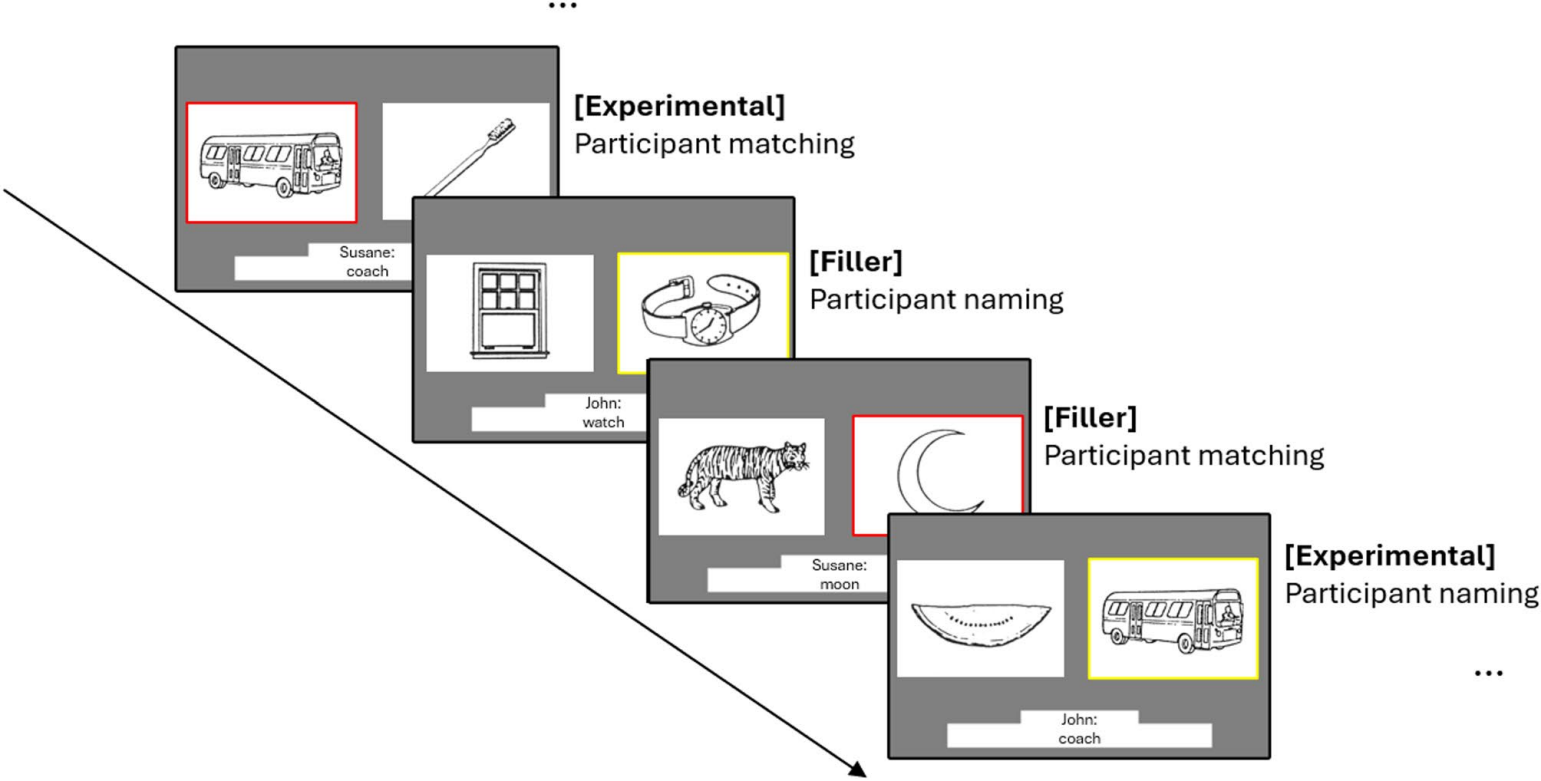
Trial sequence, showing alternating picture-matching and picture-naming trials and two filler trials intervening between each linked pair of experimental trials.

Eighteen experimental items were selected from Branigan et al.^27^; each has a favoured and a disfavoured name. For example, *bus* was favoured by more than 80% of participants in a norming task^27^, while *coach* was disfavoured. Ten practice trials (5 matching and 5 naming trials) were presented to familiarise participants with the procedure. Session 1 included 36 experimental items (18 matching and 18 naming trials for each item) and 126 filler items (63 matching and 63 naming trials). Filler items were also taken from Branigan et al.^27^ and all had one dominant name. Trials were organised into six blocks of 27, which were presented in a fixed pseudo-randomised order that ensured that two filler items were presented between each matching-naming experimental item combination (e.g. experimental matching trial– filler naming trial – filler matching trial – experimental naming trial). In each of these linked combinations the matching trial can be seen as a priming trial, providing their partner’s “choice” of term for the experimental item, and the naming trial that followed after two fillers made use of the same image as a target to examine any alignment effect. Session 2 included the same 18 experimental items to measure the continued lexical alignment effect from Session 1 (18 naming trials for each item; matching trials were not included for experimental items to examine carry-over effects of disfavoured words from Session 1) and 60 filler trials (39 matching and 21 naming trials to balance the total number of matching and naming trials). Trials were organised into three blocks of 26 items. Figure 3 illustrates the trial sequence.

In both versions of the task, items were organised into two lists, so that each list contained nine favoured and nine disfavoured experimental items; each item appeared in each condition across the two counterbalanced lists. In addition, the order of experimental and filler items between experimental items was randomised across four sub-lists. Each sub-list was then presented in a fixed order, so there were 8 fixed lists in total (2 lists × 4 sub-lists).

### Design

The study employed a 2 (Group) × 2 (Session) mixed design. Group was manipulated between subjects across two levels (wake vs. sleep) and Session was manipulated within subjects across two levels (1 vs. 2).

### Procedure

In Session 1, participants individually completed a questionnaire on Qualtrics, which included demographic information (gender, age and ethnicity) and sleep quality measures (PSQI, ESS, rMEQ and SSS). Then, pairs of participants sat opposite each other and completed a 5-minute problem-solving task (jointly discussing possible solutions to real-world problems^36^ as a “warm-up” activity that established a social relationship between the pairs of unfamiliar participants. Next, they returned to individual computers to complete the lexical alignment task in Pavlovia and finally returned to Qualtrics to complete the three social outcome measures (familiarity, perceived common ground and likeability).

The same pair of participants returned to the lab for Session 2 within 12 h; they were instructed not to consume any alcohol between the two sessions and to have a normal night of sleep, and participants in the Wake group were instructed not to take a nap. Session 2 began with the SSS questionnaire on Qualtrics, then participants completed the lexical alignment task. Finally, they completed three social outcome measures in Qualtrics.

## Data Availability

All hypotheses, methodological procedures and analysis plan were pre-registered on Open Science Framework (OSF; see https://osf.io/vkxct). All data and analysis scripts are available at https://osf.io/s3cpz.
